# Seed interior microbiome of rice genotypes indigenous to three agroecosystems of Indo-Burma biodiversity hotspot

**DOI:** 10.1186/s12864-019-6334-5

**Published:** 2019-12-03

**Authors:** Garima Raj, Mohammad Shadab, Sujata Deka, Manashi Das, Jilmil Baruah, Rupjyoti Bharali, Narayan C. Talukdar

**Affiliations:** 1 Biodiversity and Ecosystem Research, Institute of Advanced Study in Science and Technology, Guwahati, Assam, 781035 India; 2 Department of Biotechnology, Gauhati University, Guwahati, Assam, 781014 India

**Keywords:** Agroecosystem, Rice endophytes, Seed microbiome, Standing water, Deep-water, Midland, Upland

## Abstract

**Background:**

Seeds of plants are a confirmation of their next generation and come associated with a unique microbia community. Vertical transmission of this microbiota signifies the importance of these organisms for a healthy seedling and thus a healthier next generation for both symbionts. Seed endophytic bacterial community composition is guided by plant genotype and many environmental factors. In north-east India, within a narrow geographical region, several indigenous rice genotypes are cultivated across broad agroecosystems having standing water in fields ranging from 0-2 m during their peak growth stage. Here we tried to trap the effect of rice genotypes and agroecosystems where they are cultivated on the rice seed microbiota. We used culturable and metagenomics approaches to explore the seed endophytic bacterial diversity of seven rice genotypes (8 replicate hills) grown across three agroecosystems.

**Results:**

From seven growth media, 16 different species of culturable EB were isolated. A predictive metabolic pathway analysis of the EB showed the presence of many plant growth promoting traits such as siroheme synthesis, nitrate reduction, phosphate acquisition, etc. Vitamin B12 biosynthesis restricted to bacteria and archaea; pathways were also detected in the EB of two landraces. Analysis of 522,134 filtered metagenomic sequencing reads obtained from seed samples (n=56) gave 4061 OTUs. Alpha diversity indices showed significant differences in observed OTU richness (*P*≤0.05) across genotypes. Significant differences were also found between the individual hills of a rice genotype. PCoA analysis exhibited three separate clusters and revealed the clusters separated based on genotype, while agroecosystem showed a minimal effect on the variation of seed microbiota (adonis, *R*^2^=0.07, P=0.024). Interestingly, animal gut resident bacteria such as *Bifidobacterium, Faecalibacterium, Lactobacillus*, etc. were found in abundance as members of the seed microbiota.

**Conclusion:**

Overall, our study demonstrates, indigenous rice genotypes of north-east India have a unique blend of endophytic bacteria in their mature seeds. While there are notable variations among plants of the same genotype, we found similarities among genotypes cultivated in completely different environmental conditions. The beta diversity variations across the seven rice genotypes were significantly shaped by their genotype rather than their agroecosystems.

## Background

The occurrence of bacteria in the rhizosphere and internal parts of plants is a natural phenomenon [1–4]. Several studies have reported the significant role of rhizospheric and endophytic bacteria in plant growth and development [5]. Many factors drive the rhizospheric and endophytic bacterial community composition (REBCC). Rhizodeposits, host genotype, crop domestication [5], soil type [6], plant physiology, agricultural management [7], etc. are some of the many such determinants. In sexually reproducing plants, genetic information is transmitted from parent to progeny through its seeds. The information passed vertically is not only from its genome but also the metagenome, which includes DNA of endophytic microorganisms transferred to the seeds from its parent. Hardoim et al. [7] reported that in the absence of soil or any other environmental microflora, endophytes in seeds shape the plant microflora. This seed endophytic community is comprised of many bacterial species, and they display multiple functionalities [7–9]. As seeds germinate, they absorb water and activate their metabolic processes which may attract both beneficial and harmful microorganisms present in the soil. The resident endophytes may protect germinating seeds from pathogenic organisms [10] and also promote seedling development. Since the endophytic community is an essential and inevitable part of plants, their assortment from the diverse microbial community in its environment may also be stringent. For example, Compant et al. [4] reported that species diversity of the endophytic community in plants is comparatively less and is a subset of their rhizosphere microflora. Several studies have reported the population and community composition of endophytic bacteria (EB) in rice plants of different genotypes adapted to distinct environments. Among such studies, Hardoim et al. [11] found that roots or shoots of nine different rice cultivars grown under similar substrate conditions had a specific fraction of bacterial communities common to all.

Northeast India is one of the centers of origin of rice *(Oryza sativa)*, and this region is home to many local landraces of rice. The cultivated rice has originated from its wild ancestors 10,000 years ago based on evidence gathered from studies using phylogeographic approach [12, 13]. Rice plants have adapted to broad ecological conditions associated with diverse habitats used for their cultivation by human settlements. In several south-east Asian countries, such as Thailand, Myanmar and Bangladesh, rice cultivation is done in fields with no standing water to 10 to >100 cm water depth.

In NE India, rice landraces are known based on their adaptation to different agroecosystems [14, 15]. There are three well-recognized landrace groups and this grouping is based on water level in the agricultural fields where these rice complete their life cycle. Accordingly, these are delineated into deep-water (>100 cm), midland (10 - 30 cm), upland (no standing water) rice and their cultivation span between an altitude of 50 m to 7000 m msl within NE India [13, 14]. These rice varieties/landraces are stringent to their environmental requirement and do not grow well across agroecosystems. Environmental variables that may be associated with this stringency are soil, standing water depth, temperature and humidity. Microbial population of soil in these agroecosystems may also be regulated by these environmental variables and the soil is likely to harbor different bacterial communities that may contribute to seed endophytic community. Therefore, seeds of different rice genotypes might reflect a set of bacteria peculiar to their genotypes transmitted vertically and another set acquired from soil of the agroecosystem. There have not been any studies to compare microbial community in soil, water and interior of rice plant parts including seeds of field-grown rice genotypes adapted and cultivated in these agroecosystems with varying standing water depths within a narrow geographical boundary of northeast India. The objectives of this study were to (i) determine bacterial community by culture-dependent and independent techniques in seeds of rice genotypes which are very specific to an agroecosystem and are well recognized by farmers and (ii) carry out functional sequence analysis of these EB isolates.

## Results

### Population of EB

**Fig. 1:**
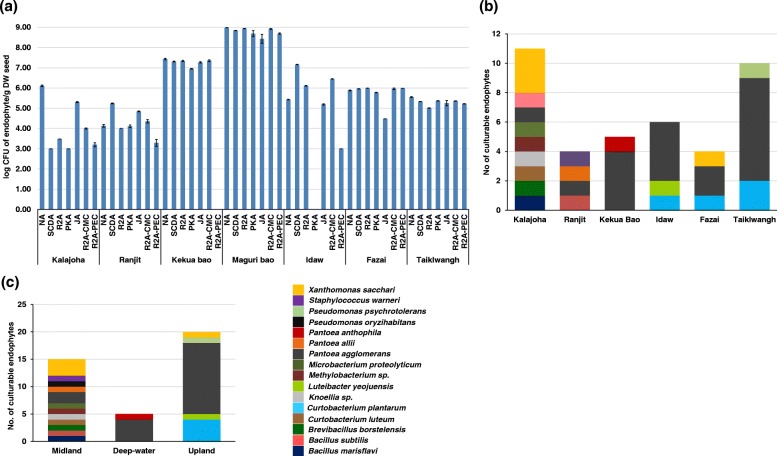
**a** Log CFU of EB in 1 gram dry weight (DW) of rice seeds across seven culture media; NA- nutrient agar, SCDA- soybean casein dextrose agar, as general purpose media, R2A- reasoner’s 2 agar for oligotrophs, PKA- pikovskaya’s agar for phosphate solubilizers, JA- jensen’s agar for nitrogen fixers, R2A/CMC- reasoner’s 2 agar/ carboxymethyl cellulose for cellulolytic bacteria, R2A/PEC- reasoner’s 2 agar/citrus pectin for pectinolytic bacteria. The error bar represents log ± S.E. distribution of EB across **b** the three agroecosystems and **c** six different rice genotypes. Stacked bars represent the diversity of EB species in mature rice seeds and the height of individual color bar shows the relative frequency of a species

The population range of aerobic EB (log cfu/g) in dry seeds (DS) across the seven media was different depending upon the genotypes of rice. EB population in different media from *Maguri bao* seeds ranged from 5.59-8.98, followed by *Kekua bao* (6.95-7.43) in different media except in R2A-PEC. The population of EB from hill landraces *Idaw, Fazai*, and *Taiklwangh* ranged from 3.00-7.16 cfu/g DS in different media, except on PKA no colony was detected from *Idaw*. Range of log cfu/g DS of EB from *Kalajoha* and *Ranjit* was lowest (3.00-6.11) among the genotypes (Fig. 1a).

### Identification, phenotype and metabolic function of EB

**Fig. 2:**
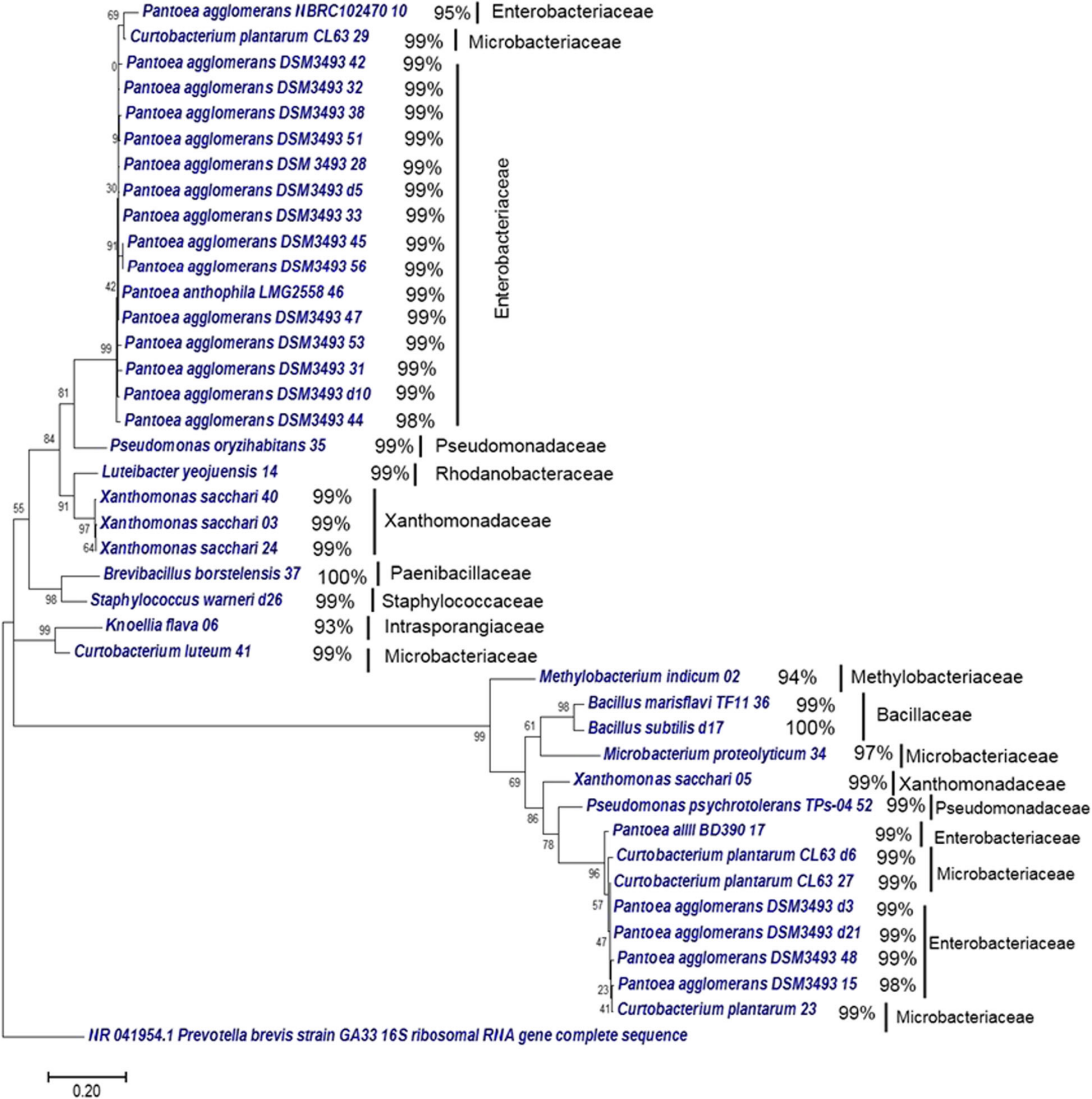
Phylogenetic relationship among the culturable EB was inferred by constructing maximum likelihood tree with 1000 bootstraps. The branch length represents 0.20 nucleotide substitutions per site, it was calculated by Tamura-Nei method using the 16S rRNA gene sequences. Tree was created with MEGA7 software. Percent similarity of the sequences obtained by aligning against NCBI 16S rDNA nr database is also represented. Families to which these bacterial species belong are also shown

The identity of randomly selected 40 isolates based on their 16S rRNA gene sequences is provided in Additional file 1: Table S1. The 16S rRNA gene sequences of isolates from six genotypes (sequences from *Maguri bao* were poor quality) were submitted to GenBank with accession numbers KY486204-KY486232, KY019244-KY019246, KY013009 - KY013011 and KY003114. Based on alignment against NCBI database, the 40 sequences were found to be representative of 3 phyla, 11 genera, and 16 different species. Distribution of the 16 species within genotypes and agroecosystems is shown in Fig. 1b,c. *Kalajoha* seeds contained highest (9) and *Kekua bao* had lowest (2) number of species (Fig. 1c). Among agroecosystems, midland contained highest (12) and deep-water had lowest (2) number of species (Fig. 1b). *Proteobacteria* was the most dominant phylum comprising five genera, eight species and 72% sequences, followed by *Actinobacteria* comprising three genera, four species and 18% sequences and *Firmicutes* comprising three genera and four species and only 10% sequences. 19 out of 40 isolates were 98-99% similar to *Pantoea agglomerans* and were detected in all the seven growth media from surface sterilized seeds of the six genotypes. Phylogenetic relationship among the 40 isolates is shown in (Fig. 2). We found that the culturable EB population of midland agroecosystem is more phylogenetically diverse than upland and deep-water agroecosystem. Same is also true for genotype *Kalajoha* than the remaining six genotypes. *Brevibacillus borstelensis 37, Knoellia flava 06* and *Curtobacterium luteum 41* of families *Paenibacillaceae, Intrasporangiaceae* and *Microbacteriaceae*, isolated from *Kalajoha* are clumped together in a clade. Similarly, *Xanthomonas sacchari 05, Microbacterium proteolyticum 34* and *Bacillus marisflavi 36*, also isolated from *Kalajoha* and belonging to distinct families cluster in a clade (Fig. 2).

**Fig. 3:**
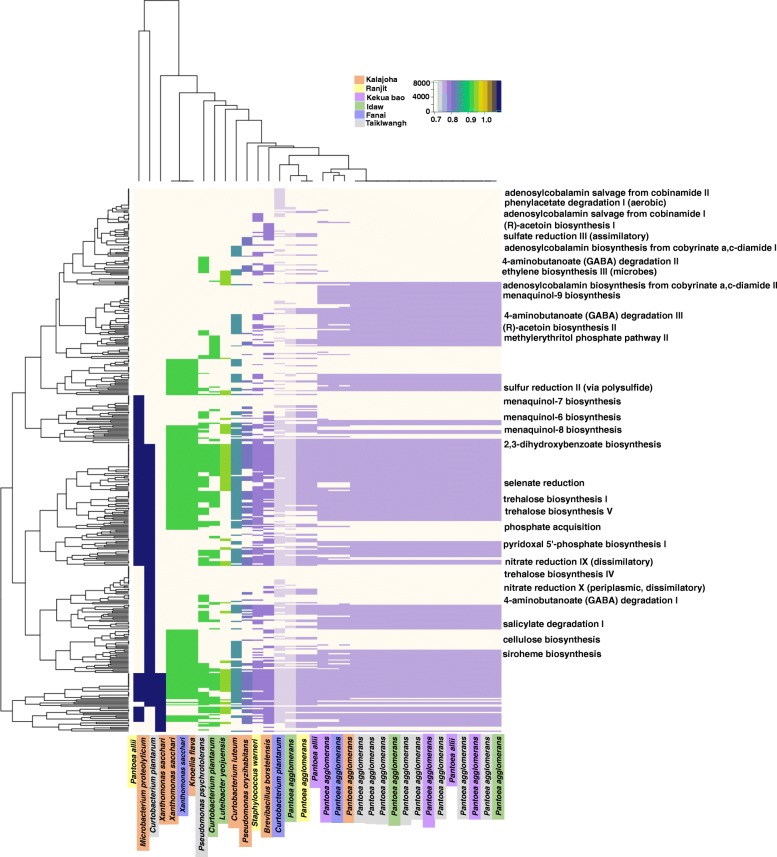
Rice plants are benefitted by different metabolic pathways of the seed EB. PAPRICA (pathway prediction by phylogenetic placement) analysis assigned 436 metabolic pathways to 34 EB isolated from seeds of the six rice genotypes. A few potentially beneficial pathways are shown in the figure. Heatmap displays presence of these metabolic pathways in the EB. Euclidean distance was calculated using "dist" function and hierarchical clustering was done using "hclust" method from heatmap.2 function of gplots package in R. A value of two was added to the data for normalization and converted to log scale. The color key shows a gradual increase in confidence score of the metabolic pathways in log scale

A total of 436 metabolic pathways were assigned based on database search for the 40 EB of the seeds. There were a large number of assigned central pathways of which several pathways are for growth and development of host plant. These pathways were found to vary in their abundance (number of times a pathway was present in an agroecosystem) depending upon agroecosystem. For example, (R)-acetoin biosynthesis I& II, responsible for the synthesis of plant beneficial volatile organic compound (VOC), acetoin [16] was found to be present in most of the EB isolated from landraces of upland agroecosystem. Similarly, pathways for siderophore production such as 2,3-dihydroxybenzoate biosynthesis and siroheme biosynthesis were also found more prominent in isolates of upland agroecosystem followed by midland agroecosystem. These two pathways had very low confidence score (0.714) in the deep-water agroecosystem. In contrast, three pathways for 4-aminobutanoate (GABA) degradation were found to be present in all the isolates of the deep-water agroecosystem. Interestingly, adenosylcobalamin (vitamin B12) biosynthesis pathways were assigned to isolates of the midland genotype *Kalajoha* and upland genotypes *Idaw* and *Taiklwangh*. Selenate reduction pathway was assigned with a higher confidence score (4.22) to the EB of midland agroecosystem followed by those in upland and deep-water (Fig. 3). A full list of the pathways with their confidence score values is given in Additional file 2: Table S2.

### General features of sequences and distribution of taxa

522,134 combined reads were obtained after quality filtering and chimera removal with an average of 9323 reads per sample (min=366, max=45574, SD=11608). 4061 OTUs were obtained across the 56 samples (7 genotypes x 8 hills) which were assigned to 29 phyla and 291 genera. *Proteobacteria, Actinobacteria, Bacteroides* and *Firmicutes* were the most dominant phyla (relative abundance >3%) covering 98.8% of total sequences. *Achromobacter, Agrobacterium, Bifidobacterium, Erwinia, Microbacterium, Ochrobactrum, Pseudomonas, Sphingomonas* and *Xanthomonas* were the nine most prevailing genera (relative abundance >2%) accounting for 74.4% of total reads.

### Assembly pattern of taxa at individual and population level

**Fig. 4:**
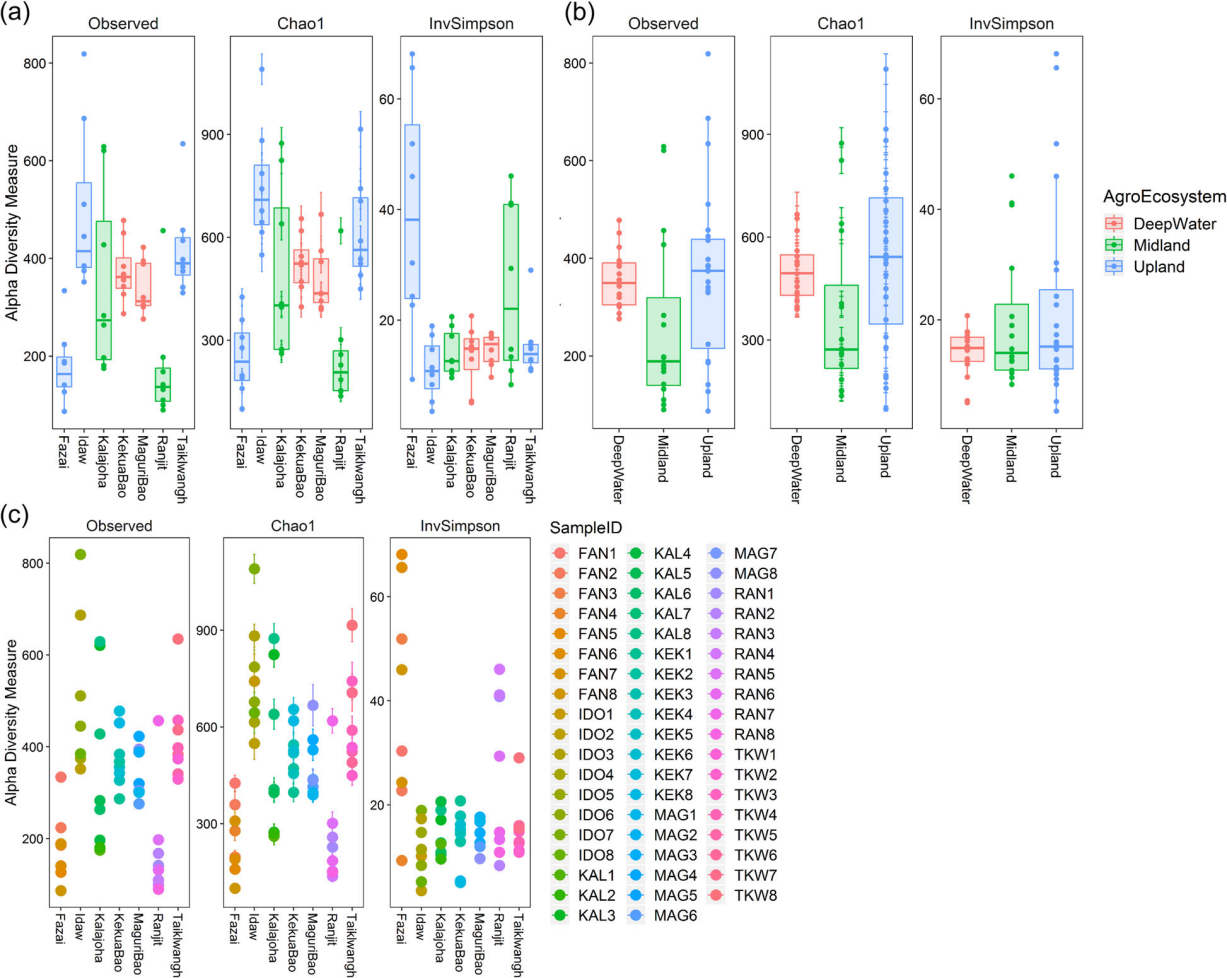
Alpha diversity of seed endosphere bacteria in **a** seven genotypes of the three agroecosystems, **b** individual hills of the genotypes. Three different methods were used to compare alpha diversity viz. observed OTUs, Chao1 and InvSimpson. Color code represents **a** three agroecosystems, **b** individual hills of a genotype

The most dominant among the 29 detected phyla with their abundance values and genera within each phyla with relative abundance >0.7% are shown in Additional file 3: Table S3. Alpha diversity of EB was measured by observed OTU richness, Simpson’s reciprocal index, and Chao1 index (Fig. 4). Diversity indices range of the EB in the seven genotypes is shown in Additional file 4: Table S4. The observed OTU richness was significantly higher in *Idaw* (1985) than in the other six genotypes as tested by student’s t-test (*P*≤0.05). It was followed by *Taiklwangh* (1701), *Kekua bao* (1597), *Kalajoha* (1509), *Maguri bao* (1427), *Ranjit* (949) and *Fazai* (896). Chao1 index was also significantly higher in *Idaw* than in the remaining genotypes (*P*≤0.05) and was followed by *Kekua bao*, *Taiklwangh*, *Kalajoha*, *Maguri bao*, *Ranjit* and *Fazai*. On the contrary, Simpson’s reciprocal index was significantly higher (*P* < 0.05) in *Fazai*, followed by *Ranjit*, *Taiklwangh*, *Maguri bao*, *Kalajoha*, *Kekua bao* and *Idaw*. Number of EB phyla inside seeds of different genotypes varied. *Kalajoha* had the highest (22), followed by *Idaw* (21), *Taiklwangh* (20), *Kekua bao* (20), *Maguri bao* (19), *Fazai* (16) and *Ranjit* (14). Similarly, total number of genera inside seed of the genotypes also varied. Of the 291 identified genera, *Kekua bao* had the highest (178), followed by *Kalajoha* (174), *Idaw* (165), *Maguri bao* (158), *Taiklwangh* (153), *Fazai* (130) and *Ranjit* (127). Irrespective of genotype, phyla *Proteobacteria, Actinobacteria, Bacteroides* and *Firmicutes* were profuse throughout the seed EB population. The most abundant *Proteobacteria* included *Pseudomonas* and *Agrobacterium* as the prevailing genera (relative abundance >0.7%) found in all the seven genotypes. The number of identified species within each of the four phyla ranged from 45-65. Unassigned OTUs were also present in a substantial number in the landraces (19.79-43.44%) (Additional file 3: Table S3). There was significant difference (ANOVA, F=8.95, *F*_*crit*_=2.18, p=2.32E-07) in bacterial community at OTU level between seeds of the seven genotypes of the three agroecosystems.

**Fig. 5:**
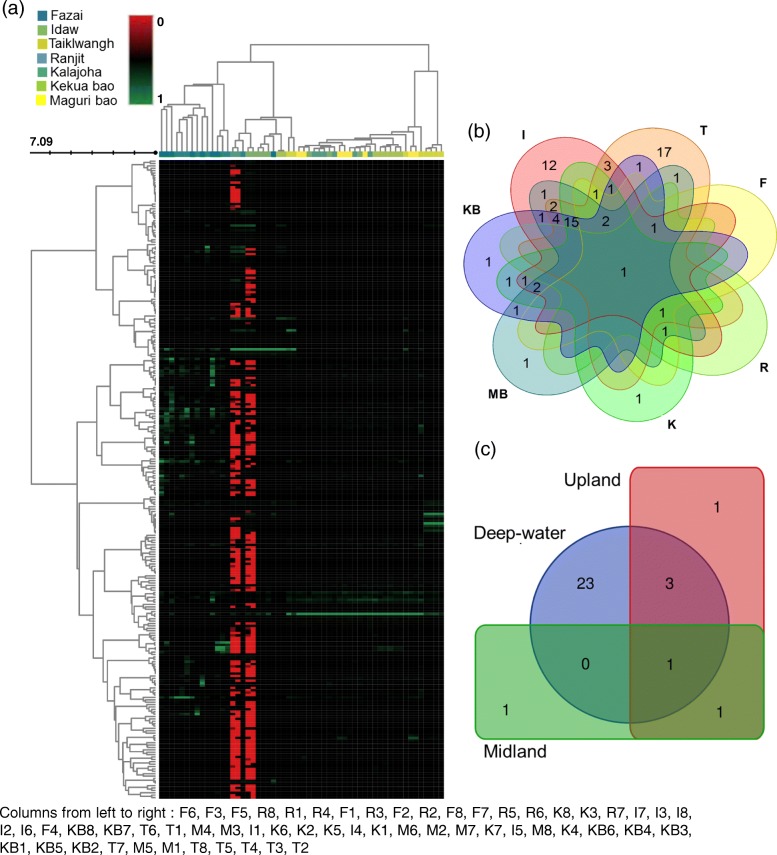
**a** Heat map of top OTUs based on high variation among the individual hills of the seven genotypes. Each color in the legend represents a genotype. Name of the columns are given at the bottom of figure. Venn diagram showing the core EB flora in **b** seven rice genotypes (F- *Fazai*, I- *Idaw*, K- *Kalajoha*, KB- *Kekua bao*, MB- *Maguri bao*, T- *Taiklwangh*), and **c** three agroecosystems based on the presence of OTUs in 100% of the samples in a group

Apart from variations between the seven genotypes, we found differences in EB taxa in rice seeds of eight replicate hills sampled within each of the genotypes. The eight replicate plants of each rice genotype had dissimilar EB community composition and abundance. Only a few members of total genera were shared by the seeds of sampled eight hills of each genotype. For example, *Kekua bao*, *Idaw*, *Taiklwangh*, *Maguri bao*, *Kalajoha*, *Fazai*, and *Ranjit* has 18, 18, 18, 15, 14, 7 and 4 common genera respectively in each of their hills and thus forming their core seed EB flora. Among the core OTUs of each genotype, there were members unique to a genotype and members present also in the remaining genotypes. We found *Taiklwangh* with the most number of unique core OTUs (17), followed by *Idaw*, *Kekua bao*, *Maguri bao*, *Kalajoha* with only one unique core OTU in each of them. Genotype *Fazai* and *Ranjit*, on the contrary had no unique core OTUs (Fig. 5b). List of OTUs detected in the eight hills of each genotype is shown in Additional file 5: Table S5. A heat map analysis of top OTUs across the individual hills of a genotype with hierarchical clustering shows relatedness in seed EB diversity among the eight hills of the seven rice genotypes. Interestingly we found, genotype *Fazai* and *Ranjit* formed a single cluster and there were similarities among their hills (Fig. 5a).

There were additional rare genera (relative abundance <0.7%) ranging from 105 (*Fazai*) to 162 (*Kekua bao*) in number which formed a distinctive mix in each of the 8 hill seeds of the seven genotypes. We found significant differences in OTU richness, chao1 index, and Simpson’s reciprocal index among the eight hills of each genotype (Fig. 4, Additional file 4: Table S4). Analysis of variance at OTU level of the 8 sampled hills showed that there was no significant difference (ANOVA, F=2.38, *F*_*crit*_=4.44, p=0.001) in EB community within the genotypes.

**Fig. 6:**
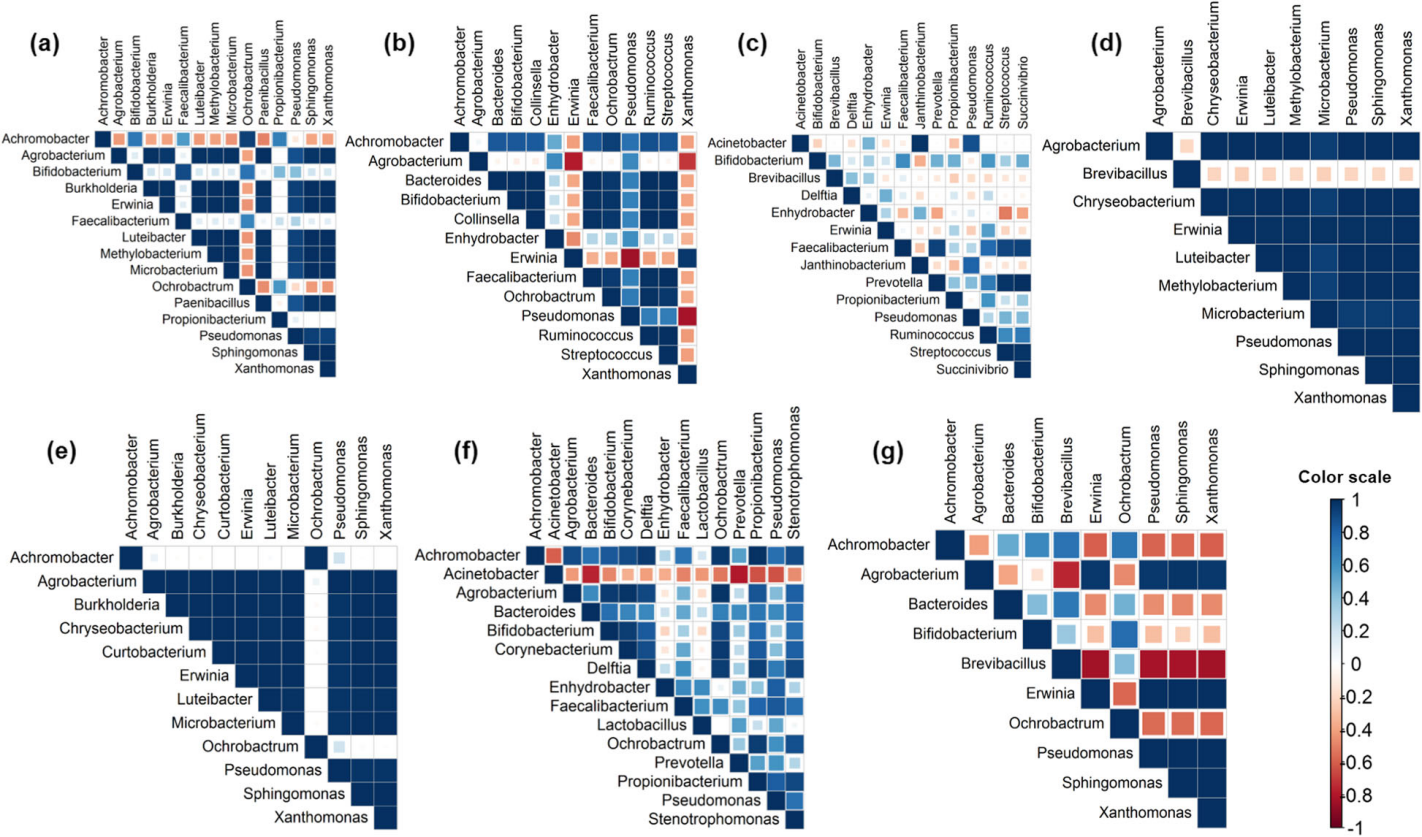
Pearson’s correlation analysis among the most abundant EB (relative abundance >1%) at genus level in the seven genotypes. **a**-*Idaw*, **b**-*Taiklwangh*, **c**-*Fazai*, **d**-*Ranjit*, **e**-*Kalajoha*, **f**-*Maguri bao* and **g**-*Kekua bao*. Color scale represents range of Pearson’s r from -1 to +1

Pearson’s correlation analysis of the most abundant EB at genus level (relative abundance >1%) among the eight hills of each genotype was performed. In *Idaw*, while *Achromobacter* was the most dominant in six hills, *Erwinia* was dominant in the remaining two hills (IDO3 and IDO7) and they were negatively correlated (r = -0.4; Fig. 6a). In *TaiklwanghErwinia* was negatively correlated with most genera except *Xanthomonas* (r = 0.96) (Fig. 6b). In *Fazai*, the dominant *Prevotella* showed a strong positive correlation with *Succinivibrio* and *Streptococcus* (r=0.99) (Fig. 6c). In *Ranjit* there was a positive correlation between all genera except *Brevibacillus* (Fig. 6d). In *Kalajoha*, the most abundant *Erwinia* displayed positive correlation with all except *Achromobacter* and *Ochrobactrum*, with which it showed no correlation (Fig. 6e). In *Maguri bao*, *Achromobacter* was positively correlated to most genera except *Acinetobacter* (r=-0.6) (Fig. 6f). In *Kekua bao*, *Pseudomonas*, the most dominant organism has strong positive relation with *Agrobacterium* (r=0.96) and *Erwinia* (r=0.99) only (Fig. 6g).

### Differential abundance of EB

**Fig. 7:**
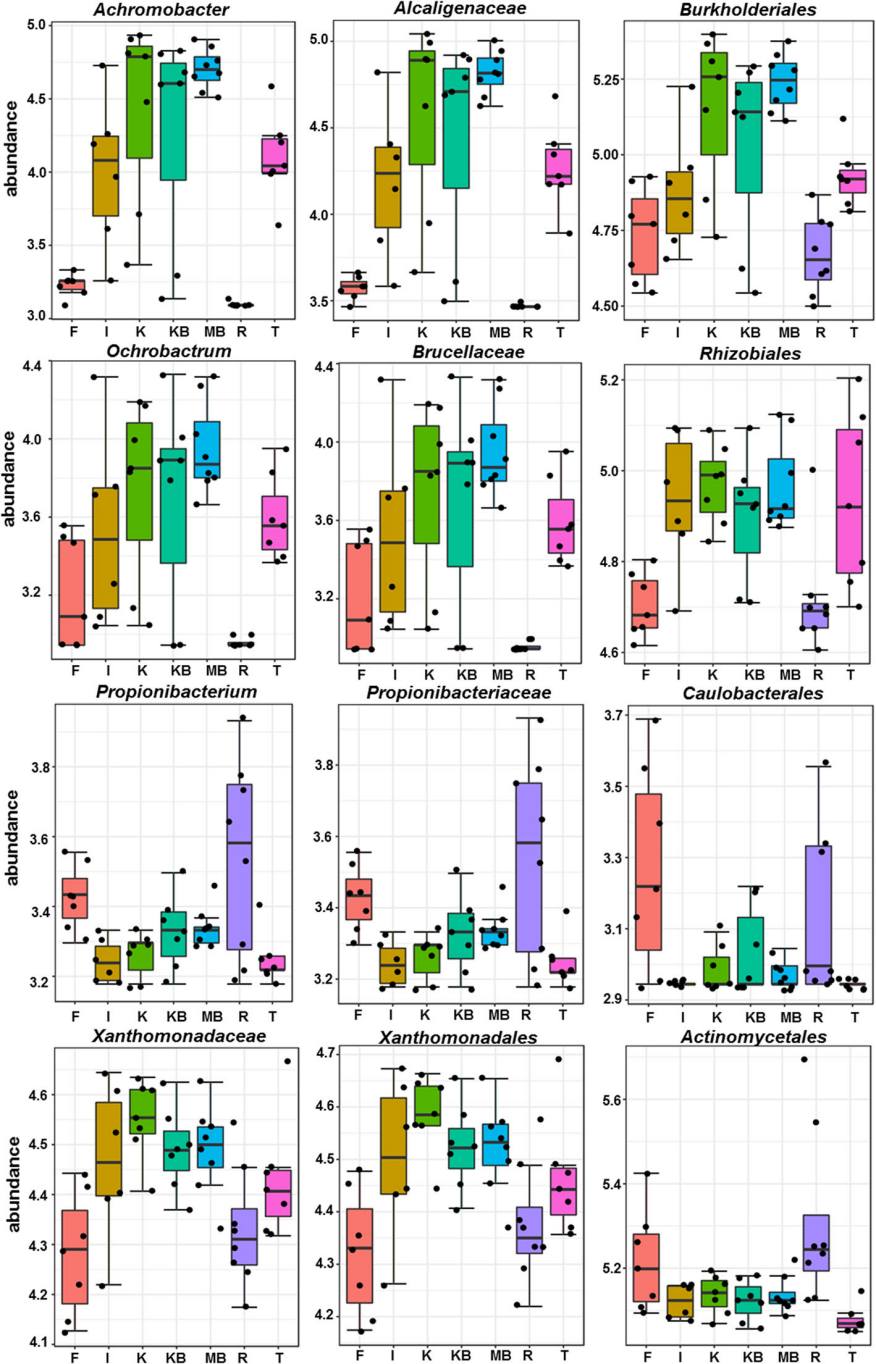
Differential abundance of rice seed endospheric taxa was estimated by t-test/ANOVA at *P*_*FDR*_ <0.05. Significant differences in abundance (*log*_10_) of EB taxa at genus, family and order level were observed across the seven rice genotypes (F- *Fazai*, I- *Idaw*, K- *Kalajoha*, KB- *Kekua bao*, MB- *Maguri bao*, T- *Taiklwangh*)

A few members of the rice seed microbiota showed an interesting pattern of their distribution among the seven genotypes. Significant differences in their population were determined by t-test/ANOVA at FDR corrected P-value cutoff of 0.05. *Planctomycetes* was the only phylum present at notably distinct numbers (ANOVA, F=4.18, *P*_*FDR*_=0.043) across the seeds of seven genotypes. Its population was lowest in *Fazai* and *Ranjit* as compared to the remaining genotypes. At the class level *Betaproteobacteria* showed significant variation in abundance (ANOVA, F=6.95, *P*_*FDR*_=0.001) (not shown in figure). Orders *Actinomycetales, Burkholderiales, Caulobacterales, Rhizobiales*, families *Alcaligenaceae, Brucellaceae, Propionibacteriaceae, Xanthomonadaceae* and genera *Achromobacter, Ochrobactrum* and *Propionibacterium* are among the other taxa differentially abundant across the genotypes (Fig. 7). We could not detect any significant OTUs differentially abundant across the agroecosystems.

### Agroecosystem effects on rice seed EB diversity

**Fig. 8:**
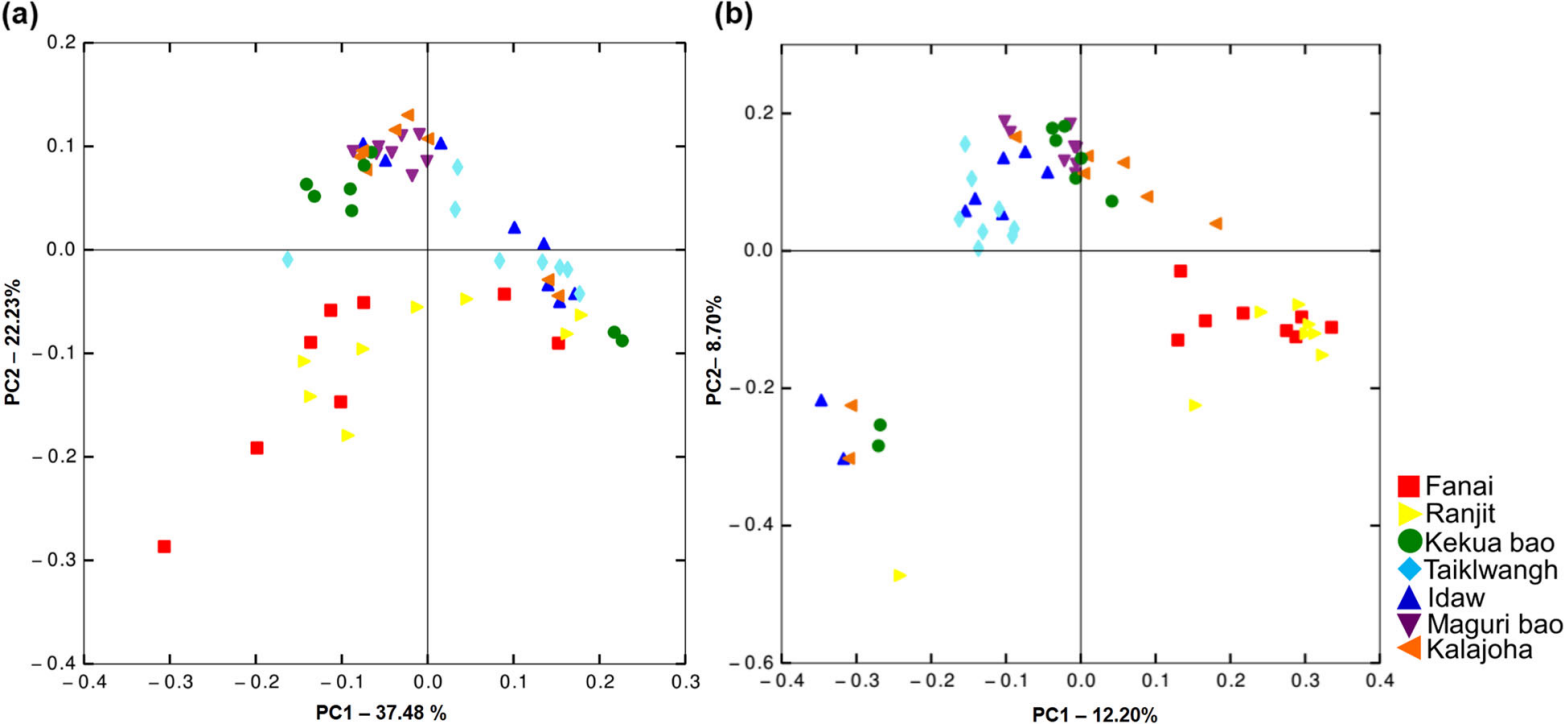
PCoA of **a** weighted unifrac, **b** binary Sorensen’s dice index showing differences and similarities of EB community in mature rice seeds of the seven genotypes (adonis, *R*^2^=0.28, p<0.001)

Among the three agroecosystems, highest number of unique OTUs was found in upland (2979), followed by deep-water (2366) and midland (1912). In terms of OTU richness and diversity, upland agroecosystem was richest and most diverse (D=0.96) followed by midland (D=0.95) and deep-water (D=0.93). The upland landrace *Fazai* and midland variety *Ranjit* formed a single cluster in the principal coordinate analysis (PCoA) plot obtained using weighted unifrac distance and binary sorensen’s dice index (Fig. 8). Beta diversity analysis clearly showed overlap in seed EB diversity across agroecosystems. This was further confirmed by ANOSIM (R=0.37, p<0.001) and adonis (*R*^2^=0.28, p<0.001). Phylogenetic distance of the seed EB diversity showed similarity in EB community of genotypes *Kekua bao, Maguri bao, Kalajoha* and *Idaw* (Fig. 8a).

There were 27 unique OTUs common in the two landraces i.e., *Kekua bao* and *Maguri bao* forming the core community of deep-water agroecosystem. This comprised of *Pseudomonas, Agrobacterium, Achromobacter, Bifidobacterium, Erwinia* and three unidentified genera of family *Xanthomonadaceae, AlcaligenaceaeEnterobacteriaceae*. Inside the upland agroecosystem landrace seeds, 6 OTUs belonging to phyla *Actinobacteria* and *Proteobacteria* formed the core microbiota. Inside seeds of all representative genotypes of midland agroecosystem (100%),only 3 OTUs belonging to genera *Pseudomonas* and *Delftia* were found to form the core EB (Fig. 5c). However when 90% genotype replicate samples were considered, 8 OTUs belonging to genera *Pseudomonas, Delftia*, *Stenotrophomonas*, *Propionibacterium* and *Enhydrobacter* were found to form the core microflora.

## Discussion

Rice seeds are associated with their unique EB microbiome. We are reporting for the first time a total EB microbiome of seeds of indigenous rice genotypes adapted to specific agroecosystem of north-east India.

Culturable approach suggests, among all the seven rice genotypes selected for this study, *Kalajoha* the traditional aromatic rice of Assam is richest in species diversity. It has 9 different EB species (Fig. 1c) representing three phyla, dominated by *Proteobacteria* (54.5%) followed by *Actinobacteria* (27.3%) and *Firmicutes* (18.2%). Whereas, collected from the same location (North Lakhimpur) and agroecosystem, the high yielding variety *Ranjit* had only four EB species, *P. allii, P. agglomerans, B. subtilis* and *S. warneri* and the community was equally shared by the two phyla, *Proteobacteria* and *Firmicutes*. Similarly, metagenomic approach based results also reveal *Kalajoha* has the second highest seed EB diversity, and *Ranjit* has lowest EB diversity among the seven genotypes. EB offers multifaceted benefits to its host plant, such as nutrient acquisition, growth promotion, production of secondary metabolites, and defence against pathogens etc., [17]. Since traditional landrace *Kalajoha* houses more diverse EB community as compared to the high yielding variety *Ranjit*, our results may suggest that the former, an aromatic landrace, demands and stores more EB to enhance its fitness in the form of nutrient acquisition, secondary metabolite production etc., as compared to the later. To further confirm this observation, studies need to be done on seed microbiome of additional numbers of high yielding rice varieties and their comparison with that of traditional landraces, cultivated in the same agroecosystem. Culturable diversity of the three upland landraces show *P. agglomerans* and *C. plantarum* as common member of their seed EB community. *L. yeojuensis, X. sacchari* and *P. psychrotolerans* however were varietal specific and were isolated from *Idaw*, *Fazai* and *Taiklwangh* respectively. A total microbiome analysis showed *Idaw* has the most heterogenous EB community followed by *Taiklwangh* and *Fazai*. Metagenome analysis showed deep-water genotype *Kekua bao* has the highest bacterial diversity at the genus level, while only two culturable species of *Pantoea* were isolated and identified from it. Both *Maguri bao* and *Kekua bao* had high numbers of unique OTUs (1427 and 1597 respectively) and the overall population of culturable EB was also highest in both landraces.

In a community different species present, interact with each other. This interaction is as a result of their phenotypic individualities. The internal plant environment i.e. community niche is necessary for nutrient acquisition, growth and development of EB, and is an important factor in shaping the endophyte community structure for their long term symbiotic association and transmission from generation to generation. This is because closely related species with similar nutritional needs are strongest competitors and thus the community comprises species which are distantly related and thus have different phenotypes [18]. As seen in phylogenetic relationship analysis of the 40 culturable isolates, EB species of different families from *Kalajoha* clustered together in a single clade and it may indicate their phenotypic attraction in the seed community to minimize competition for nutrition and survival (Fig. 2). Pearson’s correlation analysis between *Microbacterium* and *Xanthomonas* shows a strong positive relation and thus supports this observation of phenotypic attraction between the two genera (Fig. 6e).

Interestingly, *Bifidobacterium* and *Faecalibacterium* which are common and abundant residents of healthy human gut, were found among the dominant members of seed microflora in each genotype. Previously *Bifidobacterium* and *Faecalibacterium* have only been reported from rhizosphere of two native vascular plants of Antarctica, *Deschampsia antarctica* and *Colobanthus quitensis* [19]. For the first time we are reporting two abundant members of a healthy human gut microbiota as part of seed EB flora. OTUs of both genera were found across the agroecosystem and varietal barrier and surprisingly as component of core microbiota community in *Idaw, Kalajoha, Kekua bao, Maguri bao* and *Taiklwangh*. In *Fazai* and *Ranjit*, both were present in more than 50% of samples. However their lifestyle as an endophyte needs to be further evaluated, as the fields are present near human habitation and cattles graze freely, these human gut microbiota may have been sampled by the plants from soil or water.

We isolated *Microbacterium proteolyticum* from *Kalajoha*, it was first isolated from surface sterilized roots of *Halimione portulacoides* [20]. This species has not been previously reported as an endophyte of rice plants. Isolate *Luteibacter yeojuensis* was obtained from landrace *Idaw* and has a similarity of 99%. It was reported from human blood by Kampfer et al. [21]. Presence of this unusual species with high similarity in *Idaw* may indicate its adaptation as an endophyte in the landrace and a detailed study on this isolate may provide an interesting find. Although a different species of the same genus, *L. rhizovicinus* was isolated from barley rhizosphere and has been found to promote its root development [22, 23]. *Knoellia sp.* was isolated from *Kalajoha*. It was first isolated from pig manure [24]. We are reporting for the first time, the presence of this organism in the endosphere of rice. *Brevibacillus borstelensis* is a thermophilic bacteria and was isolated from *Kalajoha* with a similarity of 100%. It was found previously in, ’Marcha’- a herbal cake used as traditional starter culture to ferment local wine in north-east India and rhizosphere of wheat from northern region of India [25, 26]. But it has not been earlier reported as an endophyte of rice. We were able to identify four isolates i.e. *B. borstelensis, Knoellia sp., L. yeojuensis* and *Microbacterium proteolyticum* across the six genotypes which have till date not been found in rice endosphere. A detailed analysis of their presence as endophyte in rice seeds will further shed light into the reasons behind their choice of this habitat.

Upon identifying the EB based on their 16S rRNA gene sequences, we found that the genus *Pantoea* is consistent in the three agroecosystems as well as the six rice genotypes beyond the borders of geography, genotype and growth conditions. This indicates that the genus *Pantoea* is ubiquitous and a quintessential part of the indigenous rice genotypes of north-east India. This observation is similar to the previously reported studies on other rice varieties [27]. We observed that the population of cellulolytic, phosphate solubilizers and pectinolytic (except *Idaw* in R2A-PEC) EB increases gradually from genotypes of midland to upland to deep-water agroecosystem. However this is not the case in growth media Jensen’s agar. The rise in population of nitrogen fixing EB across the six genotypes is not agroecosystem specific. It may suggest that the population of cellulolytic, pectinolytic and phosphate solubilizing seed EB is affected by the unique agroecosystem feature i.e. the amount of standing water in rice fields and the population of nitrogen fixing EB is influenced by individual genotype.

A comparison of EB diversity of rice seed with those of other plant seeds showed striking similarities. Seed microbiome of 12 different plants from family *Brassicaceae* comprised mainly of *Proteobacteria*, *Firmicutes*,and *Actinobacteria*. Additionally they detected three OTUs belonging to genera *Pantoea, Pseudomonas*, and *Xanthomonas* present systematically in all seed samples [28]. Similarly, microbiome of seeds from 21 *Salvia miltiorrhiza* plants, a medicinal plant, collected from seven different geographic origins was predominant in *Proteobacteria*, *Bacteroidetes*, *Firmicutes*, and *Actinobacteria*, and had *Pantoea, Pseudomonas*, and *Enterobacter* as the core genera [29]. Occurence of these phyla and genera as EB across seeds of family *Brassicaceae, Poaceae* and *Lamiaceae* suggests to investigate seed microbiome of plants covering diverse families, and exploring for a core set of EB as an integral part of seeds, and their function in plants as the prokaryotic partner.

Plant scientists are yet to achieve explicit understanding on the function of seed microbiome in plant growth and development. It has been previously reported that water in deep-water paddy fields has a low concentration of major metals [30] and it may suggest that due to the low concentration of nutrients in the deep-water system, higher population of seed EB is required by the plant to ensure optimum absorption of minerals. Soil in Assam is acidic and it favours reduction of *Fe*^3+^ to *Fe*^2+^, thus causing easy and high uptake of iron. On the contrary, soil in upland fields being aerobic have iron as *Fe*^3+^ making it difficult for uptake by plants. Predictive functional analysis of the culturable isolates showed landraces of upland agroecosystem has pathways for synthesis of iron-chelating compounds in abundance followed by midland and lowest in deep-water agroecosystem. Thus showing why rice seeds from upland agroecosystem have EB with multiple and dominant pathways for synthesis of siderophores.

Rice is a major food crop of north-east India and with high nutritional value it is a benefit to the masses. Apart from plant beneficial traits we also found pathways for synthesis of many vitamins such as vitamin K2, B2, B6, B9, B12, biotin and folic acid and selenium reduction (Additional file 2: Table S2). Although these vitamins are already reported to be present in rice, but vitamin B12 has not been found in any grains, vegetables or fruits. However, a considerable amount of vitamin B12 has been detected in a soybean-based fermented food Tempe (0.7-8.0 *μ*g/100 g), a Japanese fermented beverage Batabata-cha (0.1- 1.2 *μ*g/ 100 g) etc.[31]. Vitamin B12 is a complex compound and is not required by plants. Six pathways for vitamin B12 synthesis were observed and found in traditional aromatic landrace *Kalajoha* and upland genotype *Idaw*. Adenosylcobalamin salvage from cobalamin pathway was present in all the genotypes. Since during ungerminated stage of rice seed, EB may not be actively dividing, the vitamin B12 concentration could be very little and undetectable or not present at all. But its detection in fermented foods also suggests that, if fermented, these rice landraces may also possibly show the presence of this essential nutrient.

## Conclusion

This study allowed us to clearly determine the diverse EB population that rice seeds harbor. Many studies suggests environment has an important effect on the diversity of EB. However our study shows genotype plays a more profound role in the seed EB diversity. Although cultivated in a comparatively more aerobic soil condition, we found many obligate anaerobes among the genotypes of upland agroecosystem. Interestingly, occurence of resident human gut bacteria such as *Bifidobacterium* as core member of genotypes *Kalajoha, Kekua bao, Maguri bao, Idaw*, and *Taiklwangh* show them to be well adapted to rice seed environment. However, we cannot rule out the fact that these resident human gut bacteria, might have been acquired by the host plant from its surrounding soil or water. But their mode of transmission to seeds demands further investigation. We also found, as compared to landraces, the high yielding variety *Ranjit* has low seed EB diversity, and it needs a clear understanding. The ability of these seed EB to provide beneficial metabolites, as viewed from the predictive metabolic pathway analysis, for humans, cattles and other seed grazing animals remains to be elucidated. As the current knowledge of the total diversity of indigenous rice seed endophytic flora still remains to be vastly explored, our study can provide a basis for further investigation of seed microbiome and their potential benefits.

## Methods

### Sites of rice cultivation, rice genotype and seed sample collection

Seeds from four of the seven different genotypes i.e., *Kekua bao, Maguri bao, Kalajoha* and *Ranjit* were collected from North Lakhimpur, Assam, while *Fazai, Taiklwangh* and *Idaw* [32], from Tacchip, Mizoram.*Ranjit (IET-12554) and Kekua bao* seeds were collected from fields of Regional Agricultural Research Station (RARS), North Lakhimpur. Seeds of remaining genotypes were collected directly from farmers fields and their morphological characteristics were determined based on direct observation and examination as well as interviewing the farmers. *Ranjit* is a high yielding variety and it was cultivated in field under submergence condition. Due to its high yield (40-50 q/ha), this has been a very popular variety among farmers during the last two decades in Assam. Remaining six are traditional rice landraces which farmers of the region have been growing for centuries. *Kekua bao* and *Maguri bao* are deep-water paddy grown in flood prone area with standing water which fluctuates between 1-2 m depth during its peak growth, which coincides with onset of monsoon from May-June to August-September. These landraces adapt to rising water level by rapid vertical elongation and as water recedes with cessation of monsoon during September/October, the tall plants (2-3 m) kneel up at its uppermost node where inflorescence develops. *Kalajoha* gives low yield (15-25 q/ha) but this scented rice is consumed as a delicacy item by elite which grows in fields with 10-30 cm depth of standing water. *Idaw, Fazai* and *Taiklwangh* were collected from jhum fields in hills with 50-80% gradient slope which do not experience any water stagnation during their growing period.

At the time of harvest, spanning from October to December, 2014, mature seeds of eight randomly selected rice hills were harvested directly from field, sealed in sterilized plastic bags and stored at 4^∘^C until further processing. The sampled rice genotypes were cultivated in naturally fertile land as rainfed crop and without chemical fertilizers or pesticides. Seeds were harvested from 8 randomly selected hills (plant) separately for each genotype. The 8 hills of a rice genotype were 100 cm apart from each other. Details of all genotypes are given in Table 1.

**Table 1:** Collection of rice genotypes, their habitat and location

Genotypes	Location	Environment/Habitat	Agroecosystem	GPS Coordinates	Altitude (m)
*Taiklwangh* (LR)	Location 1	Subtropical to Temperate	Upland	23.25.552N	760
				92.42.917E	
*Idaw* (LR)	Location 1	Subtropical to Temperate	Upland	23.35.465N	810
				92.43.074E	
*Fazai* (LR)	Location 1	Subtropical to Temperate	Upland	23.35.770N	721
				92.42.971E	
*Kalajoha* (LRS)	Location 2	Tropical plainland	Midland	27.14.513N	50
				94.35.670E	
*Ranjit* (HYV)	Location 2	Tropical plainland	Midland	27.14.170N	71
				94.83.871E	
*Kekua bao* (LR)	Location 2	Deep-water	Deep-water	27.14.479N	71
				94.84.053E	
*Maguri bao* (LR)	Location 2	Deep-water	Deep-water	27.14.513N	61
				94.35.670E	

(LR- Landrace, LRS- Landrace submergence, HYV- High yielding variety)

Location 1:Tacchip, Mizoram; Location 2:North Lakhimpur, Assam

### Surface sterilization, isolation of culturable EB and determination of phenotypic traits

Seeds from each of the eight hills of a genotype were pooled for isolation of culturable EB. One gram seed in triplicates was aseptically dehusked and used further in the experiment. For surface sterilization, the method of Hardoim et al. [7] was followed with modifications. Dehusked seeds were first treated with 70*%* ethanol for 30 seconds. Any remaining ethanol was washed with deionized sterile water. A cocktail of 0.1*%* sodium carbonate, 3*%* sodium chloride, 0.15*%* sodium hydroxide, and 0.2*%* (available chlorine) sodium hypochlorite was used as sterilization solution. Seeds were washed with 2*%* sodium thiosulfate pentahydrate (Merck) to remove any remaining disinfectant and then rehydrated for 1 hour in autoclaved deionized water. Effectiveness of surface sterilization method was confirmed by plating three randomly selected seeds on nutrient agar (NA) and nutrient broth (NB). Plates were incubated at 28^∘^C, and broth tubes were maintained at 28^∘^C/200 rpm and observed for five days for microbial growth if any. One gram surface sterilized seeds in triplicates were homogenized and serially diluted in 9 ml sterile deionized water. 100 *μ*l homogenate from each dilution (up to 10^−3^) was plated in triplicate on seven different culture media. These seven growth media were selected to isolate as many diverse EB as possible. Nutrient agar (general purpose media), soybean casein dextrose agar (SCDA) (general purpose media), reasoner’s 2A agar (R2A) (culturing slow growing bacteria), pikovskaya’s agar (PKA) (selective media), jensen’s agar (JA) (selective media), R2A-CMC with 0.2% CMC sodium salt/0.1% triton x-100 (selective media) and R2A-PEC with 0.2% (w/v) citrus pectin/0.1% triton X-100 (selective media) added to R2A media [11]. Agar plates were incubated at 28^∘^C and observed for bacterial growth till seven days. The number of colony forming units (cfu) from each replicate was counted under a colony counter and reported in log cfu/g dry seed (DS). Each replicate value of a dilution was multiplied with the dilution factor and then calculated for 1 gram sample. After conversion to log10 scale, mean was calculated, standard error was determined and bar graph was plotted in MS Excel (Additional file 1: Table S1). All isolates were selected based on their unique colony morphologies such as color, shape, size etc. The selected isolates were further cultured individually in agar plates for pure colonies. Growth of colony in the selective media i.e., PKA, JA, R2A-CMC and R2A-PEC was assumed to reflect the functional traits of the isolates. For example, PKA bacterial isolates with phosphate solubilization function, JA nitrogen-fixing activity, R2A-CMC and R2A-PEC media for cellulolytic and pectinolytic activities, respectively.

### DNA extraction, PCR amplification, sequencing and phylogenetic analysis

The method by Ding et al. [33] was followed with modifications. 2 ml of culture was centrifuged to pellet down cells. 567 *μ*l TE (pH-8.0), 3 *μ*l 10% SDS and 3 *μ*l proteinase K (20 mg/ml) were added and incubated for 1 hour/ 60^∘^C. 100 *μ*l 5 M NaCl was added followed by 80 *μ*l of CTAB/NaCl solution (10% CTAB in 0.7 M NaCl), mixed and incubated for 10 min/ 65^∘^C. Phenol: chloroform: isoamyl alcohol (25:24:1) was added for extraction followed by one volume of chloroform: isoamyl alcohol (24:1) to the aqueous phase. DNA was precipitated with 5 M NaCl and two volumes of chilled absolute ethanol. The pellet was washed twice with 80% ethanol. The dried pellet was resuspended in TE buffer (pH 8.0). RNase treatment was done as required. Samples were checked in agarose gel. 16S rRNA gene was amplified in Eppendorf Mastercycler using primer pair 27f (5’ - AGAGTTTGATYMTGGCTCAG) and 1492r (5’ - TACCTTGTTAYGACTT). PCR products were purified using Gen-elute PCR cleanup kit (Sigma). Purified samples were sequenced in Applied Biosystems sequencer at Xcelris Genomics, Ahmedabad, Gujarat. Two additional internal primers, 533f (5’- GTGCCAGCAGCCGCGGTAA), 805r (5’- GACTACCAGGGTATCTAATCC) were used for sequencing. Contigs were assembled based on their phred scores (>15) and identified by aligning in NCBI reference rRNA database using the blastn algorithm. 16S rRNA gene sequences were used to build a phylogenetic tree in MEGA7 software [34] to determine the relationship among them using Maximum Likelihood method with 1000 bootstrap replications and Tamura-Nei as nucleotide substitution model.*Prevotella brevis* strain GA33 was used as outgroup. Sequences from isolates of *Maguri bao* had very low phred scores and could not be identified.

### Predictive functional analysis of RNA sequences

The 16S rRNA gene sequences were individually aligned in PAPRICA (Pathway Prediction by Phylogenetic Placement) [35] pipeline to predict the metabolic pathways and putative roles of the prokaryotic endophytes. Query sequences provided to PAPRICA were searched for sequence similarity in DNA databases using Infernal. Their metabolic pathways and functions were assigned from BioCyc and MetaCyc [36] databases using pathway tools [37] through phylogenetic placement of the query sequences on a reference tree by pplacer. Whether the predicted pathways are correct was further checked by scanning through the available genome sequences of the isolates. Euclidean distance was computed and hierarchical clustering was performed to the output of PAPRICA analysis using pdist and hclust functions, respectively in R [38].

### Extraction of metagenomic DNA and next generation sequencing

Metagenomic DNA was extracted from 1 gram dehusked and surface sterilized seeds of the 8 hills of seven genotypes. Each hill was treated as a biological replicate. DNA was extracted by the method of Sharma et al. [39] For next generation sequencing, V3-V4 hypervariable region of 16s rRNA gene was targeted. Library was constructed by random fragmentation of DNA and unique 16bp index sequences were ligated to each sample. Library was generated for producing 2x300 paired end reads in Illumina MiSeq platform. Sequencing was done at Macrogen Inc., South Korea.

### Data processing and bioinformatics analysis

The demultiplexed data was processed in QIIME v1.9.1 [40]. Paired-end reads were merged using fastq-join [41] and chimeric sequences were detected using VSEARCH software [42] against gold database. After quality filtering and removal of chimera, the resulting high quality sequences were clustered into operational taxonomic units (OTUs) at 97% similarity by uclust [43] and taxonomy was assigned to the representative OTUs using SILVA database. Singletons and sequences classified as mitochondria, chloroplast, archaea, cyanobacteria, and unassigned sequences were removed. After generation of OTU table bioinformatics analysis was performed in R and QIIME 1.9.1. Differential analysis was performed in MicrobiomeAnalyst by transforming data using relative log expression (RLE) [44].

## Supplementary information

**Additional file 1** **Table S1** number of colony forming units in each replicate of the seven culture mediaacross the seven genotypes and statistical analysis.

**Additional file 2** **Table S2** list of metabolic pathways and their confidence score across the culturable solates.

**Additional file 3** **Table S3** next generation sequencing based OTUs of endophytic bacteria at different taxa and percentage abundance of unaasigned OTUs.

**Additional file 4** **Table S4** range of three alpha diversity indices in the seven rice genotypes.

**Additional file 5** **Table S5**: list of OTUs detected in all the hiils of a genotype.

## Data Availability

All sequence data are available in NCBI and metagenomics data is available in MG-RAST by the ID mgp20939 and NCBI Sequence Read Archive (SRA) under accession number PRJNA529046.

## Abbreviations

ANOSIM: Analysis of similarity
ANOVA: Analysis of variance
CMC: Carboxymethyl cellulose
CTAB: Cetyl trimethylammonium bromide
DS: dry seed
DW: Dry weight
EB: Endophytic bacteria
FDR: False discovery rate
GABA: *γ*-aminobutyric acid
HYV: High yielding variety
JA: Jensen’s agar
LR: Landrace
NA: Nutrient agar
NB: Nutrient broth
OTU: Operational taxonomic unit
PAPRICA: Pathway Prediction by Phylogenetic Placement
PEC: Pectin
PKA: Pikovskaya’s agar
QIIME: Quantitative Insights Into Microbial Ecology
R2A: Reasoner’s 2A agar
REBCC: Rhizospheric and endophytic bacterial community composition
SCDA: Soybean casein dextrose agar
SDS: Sodium dodecyl sulfate
TE: Tris-EDTA
VOC: Volatile organic compound
