# Supplementary material for: Seed interior microbiome of rice genotypes indigenous to three agroecosystems of Indo-Burma biodiversity hotspot
Source: BMC Genomics. 2019 Dec 3;20:924. doi: 10.1186/s12864-019-6334-5 (PMC6892021; doi:10.1186/s12864-019-6334-5)
Supplement: Supplementary file 1 — Additional file 1 Table S1 number of colony forming units in each replicate of the seven culture mediaacross the seven genotypes and statistical analysis. [file 12864_2019_6334_MOESM1_ESM.pdf]

TABLE S1. Number of colony forming units in each replicate of the seven culture media across the seven genotypes and statistical analysis.

		CFU per replicate										
**Genotype**	**media**	**R1**	**R2**	**R3**	**mean CFU**	**CFU/g dry seed**	**Log R1**	**Log R2**	**Log R3**	**Mean**	**Std dev**	**Std error**
**Idaw**	R2A-CMC	272	291	295	286	2.8x10^6	6.43	6.46	6.47	6.45	0.02	0.012
	JA	170	160	135	155	1.5x10^5	5.23	5.20	5.13	5.19	0.05	0.030
	R2A-PEC	1	1	1	1	1.0x10^3	3.00	3.00	3.00	3.00	0.00	0.000
	NA	280	260	270	270	2.7x10^5	5.45	5.41	5.43	5.43	0.02	0.012
	SCDA	150	142	143	145	1.4x10^7	7.18	7.15	7.15	7.16	0.02	0.010
	R2A	137	123	124	128	1.2x10^6	6.14	6.10	6.10	6.11	0.02	0.013
**Kekua Bao**	NA	297	224	289	270	2.7X10^7	7.47	7.35	7.46	7.43	0.07	0.038
	SCDA	188	225	211	208	2.0X10^7	7.27	7.35	7.32	7.31	0.04	0.023
	PKA	84	96	90	90	9.0X10^6	6.92	6.98	6.95	6.95	0.03	0.017
	R2A-CMC	226	186	266	226	2.2X10^7	7.35	7.27	7.42	7.35	0.08	0.043
	R2A	215	197	233	215	2.1X10^7	7.33	7.30	7.40	7.34	0.05	0.030
	JA	182	205	159	182	1.8X10^7	7.30	7.31	7.20	7.27	0.06	0.035
**Fazai**	R2A-PEC	97	97	97	97	9.7X10^5	5.99	5.99	5.99	5.99	0.00	0.000
	R2A	100	95	99	98	9.8x10^5	6.00	5.98	5.99	5.99	0.01	0.006
	SCDA	95	91	87	91	9.1x10^5	5.98	5.96	5.94	5.96	0.02	0.012

	R2A-CMC	107	86	86	93	9.3x10^5	6.03	5.93	5.93	5.96	0.06	0.033
	PKA	61	57	65	61	6.1x10^5	5.78	5.76	5.81	5.78	0.03	0.015
	JA	3	3	3	3	3.0X10^4	4.48	4.48	4.48	4.48	0.00	0.000
	NA	70	73	76	73	7.3x10^5	5.84	5.90	5.90	5.88	0.03	0.020
**Ranjit**	NA	mat	16	12	14	1.4x10^4	4.00	4.20	4.18	4.13	0.11	0.064
	R2A	1	1	1	1	1.0x10^4	4.00	4.00	4.00	4.00	0.00	0.000
	JA	69	75	63	69	6.9x10^4	4.84	4.87	4.80	4.84	0.04	0.020
	SCDA	173	147	145	155	1.55x10^5	5.24	5.27	5.20	5.24	0.04	0.020
	PKA	10	17	12	13	1.3x10^4	4.00	4.23	4.10	4.11	0.12	0.067
	R2A-PEC	1	3	2	2	2.0x10^3	3.00	3.58	3.30	3.29	0.29	0.167
	R2A-CMC	16	25	19	20	2.0x10^4	4.20	4.49	4.38	4.36	0.15	0.085
**Taiklwangh**	NA	293	289	306	296	2.96x10^5	5.57	5.50	5.59	5.55	0.05	0.027
	R2A-CMC	228	235	221	228	2.2x10^5	5.36	5.37	5.34	5.36	0.02	0.009
	R2A-PEC	171	165	159	165	1.6x10^5	5.23	5.22	5.20	5.22	0.02	0.009
	R2A	102	105	99	102	1.02x10^5	5.01	5.02	4.99	5.01	0.02	0.009
	SCDA	221	219	211	217	2.17x10^5	5.34	5.34	5.32	5.33	0.01	0.007
	PKA	243	235	227	235	2.35x10^5	5.39	5.37	5.36	5.37	0.02	0.009
	JA	253	231	mat	242	2.42x10^5	5.40	5.36	5.00	5.25	0.22	0.127
**Kalajoha**	R2A-CMC	11	10	9	10	1.0x10^4	4.04	4.00	3.95	4.00	0.05	0.026
	PKA	1	1	1	1	1.0x10^3	3.00	3.00	3.00	3.00	0.00	0.000
	R2A	3	3	3	3	3.0x10^3	3.48	3.48	3.48	3.48	0.00	0.000

	NA	11	13	15	13	1.3x10^6	6.04	6.11	6.18	6.11	0.07	0.040
	SCDA	1	1	1	1	1.0x10^3	3.00	3.00	3.00	3.00	0.00	0.000
	R2A-PEC	2	1	2	1.7	1.7x10^3	3.30	3.00	3.30	3.20	0.17	0.100
	JA	180	200	220	200	2.0x10^5	5.25	5.30	5.34	5.30	0.05	0.026
**Maguri bao**	NA	97	95	93	95	9.5X10^8	8.99	8.98	8.97	8.98	0.01	0.006
	R2A	84	87	89	86.7	8.67X10^8	8.92	8.94	8.95	8.94	0.02	0.009
	JA	58	10	34	34	3.4X10^8	8.76	8.00	8.53	8.43	0.39	0.225
	SCDA	69	70	68	69	6.9X10^8	8.84	8.85	8.83	8.84	0.01	0.006
	PKA	81	26	57	54.7	5.47X10^8	8.91	8.41	8.76	8.69	0.26	0.148
	R2A-PEC	45	55	47	49	4.9X10^8	8.65	8.74	8.67	8.69	0.05	0.027
	R2A-CMC	87	86	75	82.7	8.27X10^8	8.94	8.93	8.88	8.92	0.03	0.019
