# Supplementary material for: Seed interior microbiome of rice genotypes indigenous to three agroecosystems of Indo-Burma biodiversity hotspot
Source: BMC Genomics. 2019 Dec 3;20:924. doi: 10.1186/s12864-019-6334-5 (PMC6892021; doi:10.1186/s12864-019-6334-5)
Supplement: Supplementary file 3 — Additional file 3 Table S3 next generation sequencing based OTUs of endophytic bacteria at different taxa and percentage abundance of unaasigned OTUs. [file 12864_2019_6334_MOESM3_ESM.pdf]

Table S3: Next generation sequencing based OTUs of endophytic bacteria at different taxa and percentage abundance of unaasigned OTUs

Genotype and total no. of phyla	Diversity at different taxa level				Unassigned OTU abundance (%)
	Dominant phylum (relative abundace >3%)	Abundance (%)	Dominant genera within the phylum (relative abundance >0.7%)	Number of identified species	
Idaw	*Proteobacteria*	85.31	*Achromobacter* (17.02), *Erwinia* (12.99), *Xanthomonas* (10.72), *Pseudomonas* (9.06), *Agrobacterium* (6.97), *Ochrobactrum* (6.87), *Stenotrophomonas* (0.78)	65	43.44
	Actinobacteria	5.98	*Bifidobacterium* (1.2), *Propionibacterium* (1.1)		
	Firmicutes	5.18	*Faecalibacterium* (1.2)		
	Bacteroidetes	2.39	*Bacteroides* (0.99)		
Taiklwangh	*Proteobacteria*	75.88	*Achromobacter (16.0)*, *Erwinia* (32.3), *Xanthomonas* (1.5), *Pseudomonas* (6.1), *Agrobacterium* (6.5), *Ochrobactrum* (6.6), *Stenotrophomonas* (0.89), *Enhydrobacter* (0.99)	62	21.92
	Actinobacteria	8.33	*Bifidobacterium* (6.1), *Propionibacterium* (0.82)		
	Firmicutes	9.95	*Faecalibacterium* (2.2)		
	Bacteroidetes	4.41	*Bacteroides* (3.0)		
Fazai	*Proteobacteria*	52.10	*Achromobacter (0.78), Erwinia (11.3), Pseudomonas* (14.9), *Agrobacterium* (0.88), *Ochrobactrum* (0.87), *Stenotrophomonas* (0.71), *Enhydrobacter* (1.9)	45	29.18
	Actinobacteria	9.56	*Bifidobacterium* (2.2), *Propionibacterium* (2.3)		
	Firmicutes	20.35	*Faecalibacterium* (2.2)		
	Bacteroidetes	16.61	*Bacteroides* (0.97)		
Ranjit	*Proteobacteria*	86.75	*Erwinia* (33.7), *Xanthomonas* (12.4), *Pseudomonas* (9.0), *Agrobacterium* (10.4), *Enhydrobacter* (0.74)	46	30.64
	Actinobacteria	6.32	*Propionibacterium* (0.70)		
	Firmicutes	3.50			
	Bacteroidetes	3.07			
Kalajoha	*Proteobacteria*	84.62	*Achromobacter (11.7), Erwinia* (21.6), *Xanthomonas (10.3), Pseudomonas* (12.5), *Agrobacterium* (7.8), *Ochrobactrum* (4.5), *Stenotrophomonas* (0.74)	59	29.51
	Actinobacteria	6.63	*Bifidobacterium* (0.71)		
	Firmicutes	3.35	*Faecalibacterium* (0.75)		
	Bacteroidetes	4.62	*Bacteroides* (0.84)		
Maguri bao	*Proteobacteria*	72.03	*Achromobacter* (34.3), *Pseudomonas* (8.4), *Agrobacterium* (4.4), *Ochrobactrum* (14.0), *Stenotrophomonas* (1.5), *Enhydrobacter* (1.0)	60	19.79
	Actinobacteria	7.81	*Bifidobacterium* (2.2), *Propionibacterium* (1.7)		
	Firmicutes	11.02	*Faecalibacterium* (1.3)		
	Bacteroidetes	5.82	*Bacteroides* (2.4)		
Kekua bao	*Proteobacteria*	87.77	*Achromobacter (13.9), Erwinia (19.5), Xanthomonas (9.8), Pseudomonas* (24.9), *Agrobacterium* (3.8), *Ochrobactrum* (5.4), *Enhydrobacter* (0.78)	59	43.1
	Actinobacteria	3.39	*Propionibacterium* (0.89), *Bifidobacterium* (1.0)		
	Firmicutes	5.58	*Faecalibacterium* (466)		
	Bacteroidetes	2.41	*Bacteroides* (1.3)		
