# Supplementary material for: Seed interior microbiome of rice genotypes indigenous to three agroecosystems of Indo-Burma biodiversity hotspot
Source: BMC Genomics. 2019 Dec 3;20:924. doi: 10.1186/s12864-019-6334-5 (PMC6892021; doi:10.1186/s12864-019-6334-5)
Supplement: Supplementary file 4 — Additional file 4 Table S4 range of three alpha diversity indices in the seven rice genotypes. [file 12864_2019_6334_MOESM4_ESM.pdf]

Table S4: Range of three alpha diversity indices in the seven rice genotypes

Genotype	Observed OTUs	InvSimpson	Chao1
Idaw	352-819	3.51-18.94	548.9-1090.4
Taiklwangh	330-635	10.89-29.05	449.7-915.1
Fanai	87-334	9.31-68.16	99.8-425.9
Ranjit	90-457	8.33-46.06	137.1-619.0
Kalajoha	175-629	9.58-20.64	261.4-874.1
Maguri bao	276-423	9.67-17.64	389.56-666.95
Kekua bao	287-478	5.04-20.77	397.3-654.4
