# Supplementary material for: Seed interior microbiome of rice genotypes indigenous to three agroecosystems of Indo-Burma biodiversity hotspot
Source: BMC Genomics. 2019 Dec 3;20:924. doi: 10.1186/s12864-019-6334-5 (PMC6892021; doi:10.1186/s12864-019-6334-5)
Supplement: Supplementary file 5 — Additional file 5 Table S5: list of OTUs detected in all the hiils of a genotype. [file 12864_2019_6334_MOESM5_ESM.pdf]

Table S5: List of OTUs detected in all the hiils of a genotype

Host genotype	Core OTUs						Total OTUs across 100% of sample
	p__Actinobacteria	*c__Actinobacteria*	*o__Bifidobacteriales*	*f__Bifidobacteriaceae*	*g__Bifidobacterium*	*s__adolescentis*	
	p__Actinobacteria	*c__Actinobacteria*	*o__Bifidobacteriales*	*f__Bifidobacteriaceae*	*g__Bifidobacterium*	*s__*	
	p__Actinobacteria	*c__Actinobacteria*	*o__Actinomycetales*	*f__Propionibacteriaceae*	*g__Propionibacterium*	*s__acnes*	
	p__Firmicutes	*c__Clostridia*	*o__Clostridiales*	*f__Ruminococcaceae*	*g__Faecalibacterium*	*s__prausnitzii*	
	p__Proteobacteria	*c__Gammaproteobacteria*	*o__Xanthomonadales*	*f__Xanthomonadaceae*	*g__*	*s__*	
	p__Proteobacteria	*c__Gammaproteobacteria*	*o__Enterobacteriales*	*f__Enterobacteriaceae*	*g__*	*s__*	
	p__Proteobacteria	*c__Betaproteobacteria*	*o__Burkholderiales*	*f__Alcaligenaceae*	*g__*	*s__*	
	p__Proteobacteria	*c__Betaproteobacteria*	*o__Burkholderiales*	*f__Alcaligenaceae*	*g__Achromobacter*	*s__*	
	p__Proteobacteria	*c__Alphaproteobacteria*	*o__Rhizobiales*	*f__Rhizobiaceae*	*g__Agrobacterium*	*s__*	
**Idaw (n=8)**	*p__Proteobacteria*	*c__Betaproteobacteria*	*o__Burkholderiales*	*f__Comamonadaceae*	*g__Delftia*	*s__*	49
	p__Proteobacteria	*c__Gammaproteobacteria*	*o__Pseudomonadales*	*f__Moraxellaceae*	*g__Enhydrobacter*	*s__*	
	p__Proteobacteria	*c__Gammaproteobacteria*	*o__Enterobacteriales*	*f__Enterobacteriaceae*	*g__Enterobacter*		
	p__Proteobacteria	*c__Gammaproteobacteria*	*o__Enterobacteriales*	*f__Enterobacteriaceae*	*g__Erwinia*		
	p__Proteobacteria	*c__Alphaproteobacteria*	*o__Rhizobiales*	*f__Brucellaceae*	*g__Ochrobactrum*	*s__*	
	p__Proteobacteria	*c__Gammaproteobacteria*	*o__Pseudomonadales*	*f__Pseudomonadaceae*	*g__Pseudomonas*	*s__*	
	p__Proteobacteria	*c__Gammaproteobacteria*	*o__Pseudomonadales*	*f__Pseudomonadaceae*	*g__Pseudomonas*	*s__viridiflava*	
	p__Proteobacteria	*c__Alphaproteobacteria*	*o__Sphingomonadales*	*f__Sphingomonadaceae*	*g__Sphingomonas*	*s__*	
	p__Proteobacteria	*c__Gammaproteobacteria*	*o__Xanthomonadales*	*f__Xanthomonadaceae*	*g__Stenotrophomonas*	*s__*	
	p__Proteobacteria	*c__Alphaproteobacteria*	*o__Rhizobiales*				
	p__Actinobacteria	*c__Actinobacteria*	*o__Bifidobacteriales*	*f__Bifidobacteriaceae*	*g__Bifidobacterium*	*s__adolescentis*	
	p__Actinobacteria	*c__Actinobacteria*	*o__Bifidobacteriales*	*f__Bifidobacteriaceae*	*g__Bifidobacterium*	*s__*	
	p__Actinobacteria	*c__Actinobacteria*	*o__Actinomycetales*	*f__Corynebacteriaceae*	*g__Corynebacterium*	*s__*	
	p__Actinobacteria	*c__Actinobacteria*	*o__Actinomycetales*	*f__Propionibacteriaceae*	*g__Propionibacterium*	*s__acnes*	
	p__Bacteroidetes	*c__Bacteroidia*	*o__Bacteroidales*	*f__Bacteroidaceae*	*g__Bacteroides*	*s__*	
	p__Firmicutes	*c__Bacilli*	*o__Turicibacterales*	*f__Turicibacteraceae*	*g__Turicibacter*	*s__*	
	p__Proteobacteria	*c__Gammaproteobacteria*	*o__Xanthomonadales*	*f__Xanthomonadaceae*	*g__*	*s__*	
	p__Proteobacteria	*c__Betaproteobacteria*	*o__Burkholderiales*	*f__Alcaligenaceae*	*g__*	*s__*	
	p__Proteobacteria	*c__Gammaproteobacteria*	*o__Enterobacteriales*	*f__Enterobacteriaceae*	*g__*	*s__*	
**Taiklwangh (n=8)**	*p__Proteobacteria*	*c__Betaproteobacteria*	*o__Burkholderiales*	*f__Alcaligenaceae*	*g__Achromobacter*	*s__*	50
	p__Proteobacteria	*c__Alphaproteobacteria*	*o__Rhizobiales*	*f__Rhizobiaceae*	*g__Agrobacterium*	*s__*	
	p__Proteobacteria	*c__Betaproteobacteria*	*o__Burkholderiales*	*f__Comamonadaceae*	*g__Comamonas*	*s__*	
	p__Proteobacteria	*c__Gammaproteobacteria*	*o__Pseudomonadales*	*f__Moraxellaceae*	*g__Enhydrobacter*	*s__*	
	p__Proteobacteria	*c__Gammaproteobacteria*	*o__Enterobacteriales*	*f__Enterobacteriaceae*	*g__Erwinia*	*s__*	
	p__Proteobacteria	*c__Alphaproteobacteria*	*o__Rhizobiales*	*f__Phyllobacteriaceae*	*g__Mesorhizobium*	*s__*	
	p__Proteobacteria	*c__Alphaproteobacteria*	*o__Rhizobiales*	*f__Brucellaceae*	*g__Ochrobactrum*	*s__*	
	p__Proteobacteria	*c__Gammaproteobacteria*	*o__Pseudomonadales*	*f__Pseudomonadaceae*	*g__Pseudomonas*	*s__*	
	p__Proteobacteria	*c__Gammaproteobacteria*	*o__Pseudomonadales*	*f__Pseudomonadaceae*	*g__Pseudomonas*	*s__viridiflava*	
	p__Proteobacteria	*c__Gammaproteobacteria*	*o__Xanthomonadales*	*f__Xanthomonadaceae*	*g__Stenotrophomonas*	*s__*	
	p__Proteobacteria	*c__Alphaproteobacteria*	*o__Rhizobiales*				
	p__Actinobacteria	*c__Actinobacteria*	*o__Actinomycetales*	*f__Propionibacteriaceae*	*g__Propionibacterium*	*s__acnes*	
	p__Proteobacteria	*c__Betaproteobacteria*	*o__Burkholderiales*	*f__Comamonadaceae*	*g__Delftia*	*s__*	
**Fanai (n=8)**	*p__Proteobacteria*	*c__Gammaproteobacteria*	*o__Pseudomonadales*	*f__Moraxellaceae*	*g__Enhydrobacter*	*s__*	7
	p__Proteobacteria	*c__Gammaproteobacteria*	*o__Pseudomonadales*	*f__Pseudomonadaceae*	*g__Pseudomonas*		

	p__Proteobacteria	*c__Gammaproteobacteria*	*o__Enterobacteriales*	*f__Enterobacteriaceae*			
	p__Proteobacteria	*c__Betaproteobacteria*	*o__Burkholderiales*	*f__Comamonadaceae*	*g__Delftia*	*s__*	
**Ranjit (n=8)**	*p__Proteobacteria*	*c__Gammaproteobacteria*	*o__Pseudomonadales*	*f__Moraxellaceae*	*g__Enhydrobacter*	*s__*	4
	p__Proteobacteria	*c__Gammaproteobacteria*	*o__Pseudomonadales*	*f__Pseudomonadaceae*	*g__Pseudomonas*		

	p__Actinobacteria	c__Actinobacteria	o__Bifidobacteriales	f__Bifidobacteriaceae	g__Bifidobacterium	s__adolescentis	
	p__Actinobacteria	c__Actinobacteria	o__Bifidobacteriales	f__Bifidobacteriaceae	g__Bifidobacterium	s__	
	p__Actinobacteria	c__Actinobacteria	o__Actinomycetales	f__Propionibacteriaceae	g__Propionibacterium	s__acnes	
	p__Firmicutes	c__Clostridia	o__Clostridiales	f__Ruminococcaceae	g__Faecalibacterium	s__prausnitzii	
	p__Proteobacteria	c__Gammaproteobacteria	o__Xanthomonadales	f__Xanthomonadaceae	g__	s__	
	p__Proteobacteria	c__Gammaproteobacteria	o__Enterobacteriales	f__Enterobacteriaceae	g__	s__	
**Kalajoha (n=8)**	p__Proteobacteria	c__Betaproteobacteria	o__Burkholderiales	f__Alcaligenaceae	g__	s__	27
	p__Proteobacteria	c__Betaproteobacteria	o__Burkholderiales	f__Alcaligenaceae	g__Achromobacter	s__	
	p__Proteobacteria	c__Alphaproteobacteria	o__Rhizobiales	f__Rhizobiaceae	g__Agrobacterium	s__	
	p__Proteobacteria	c__Betaproteobacteria	o__Burkholderiales	f__Comamonadaceae	g__Delftia	s__	
	p__Proteobacteria	c__Alphaproteobacteria	o__Rhizobiales	f__Brucellaceae	g__Ochrobactrum	s__	
	p__Proteobacteria	c__Gammaproteobacteria	o__Pseudomonadales	f__Pseudomonadaceae	g__Pseudomonas	s__	
	p__Proteobacteria	c__Gammaproteobacteria	o__Pseudomonadales	f__Pseudomonadaceae	g__Pseudomonas	s__viridiflava	
	p__Proteobacteria	c__Gammaproteobacteria	o__Xanthomonadales	f__Xanthomonadaceae	g__Stenotrophomonas	s__	
	p__Actinobacteria	c__Actinobacteria	o__Bifidobacteriales	f__Bifidobacteriaceae	g__Bifidobacterium	s__adolescentis	
	p__Actinobacteria	c__Actinobacteria	o__Bifidobacteriales	f__Bifidobacteriaceae	g__Bifidobacterium	s__	
	p__Actinobacteria	c__Actinobacteria	o__Actinomycetales	f__Brevibacteriaceae	g__Brevibacterium	s__	
	p__Actinobacteria	c__Actinobacteria	o__Actinomycetales	f__Corynebacteriaceae	g__Corynebacterium	s__	
	p__Actinobacteria	c__Actinobacteria	o__Actinomycetales	f__Propionibacteriaceae	g__Propionibacterium	s__acnes	
	p__Proteobacteria	c__Gammaproteobacteria	o__Xanthomonadales	f__Xanthomonadaceae	g__	s__	
	p__Proteobacteria	c__Betaproteobacteria	o__Burkholderiales	f__Alcaligenaceae	g__	s__	
	p__Proteobacteria	c__Betaproteobacteria	o__Burkholderiales	f__Alcaligenaceae	g__Achromobacter	s__	
**Maguri bao (n=8)**	p__Proteobacteria	c__Alphaproteobacteria	o__Rhizobiales	f__Rhizobiaceae	g__Agrobacterium	s__	34
	p__Proteobacteria	c__Betaproteobacteria	o__Burkholderiales	f__Comamonadaceae	g__Delftia	s__	
	p__Proteobacteria	c__Gammaproteobacteria	o__Pseudomonadales	f__Moraxellaceae	g__Enhydrobacter	s__	
	p__Proteobacteria	c__Gammaproteobacteria	o__Enterobacteriales	f__Enterobacteriaceae	g__Erwinia		
	p__Proteobacteria	c__Alphaproteobacteria	o__Rhizobiales	f__Brucellaceae	g__Ochrobactrum	s__	
	p__Proteobacteria	c__Gammaproteobacteria	o__Pseudomonadales	f__Pseudomonadaceae	g__Pseudomonas	s__viridiflava	
	p__Proteobacteria	c__Gammaproteobacteria	o__Pseudomonadales	f__Pseudomonadaceae	g__Pseudomonas	s__	
	p__Proteobacteria	c__Gammaproteobacteria	o__Xanthomonadales	f__Xanthomonadaceae	g__Stenotrophomonas	s__	
	p__Proteobacteria	c__Alphaproteobacteria	o__Rhizobiales				
	p__Proteobacteria	c__Gammaproteobacteria	o__Enterobacteriales	f__Enterobacteriaceae			
	p__Actinobacteria	c__Actinobacteria	o__Bifidobacteriales	f__Bifidobacteriaceae	g__Bifidobacterium	s__adolescentis	
	p__Actinobacteria	c__Actinobacteria	o__Bifidobacteriales	f__Bifidobacteriaceae	g__Bifidobacterium	s__	
	p__Actinobacteria	c__Actinobacteria	o__Actinomycetales	f__Corynebacteriaceae	g__Corynebacterium	s__	
	p__Actinobacteria	c__Actinobacteria	o__Actinomycetales	f__Propionibacteriaceae	g__Propionibacterium	s__acnes	
	p__Firmicutes	c__Clostridia	o__Clostridiales	f__Ruminococcaceae	g__Faecalibacterium	s__prausnitzii	
	p__Proteobacteria	c__Gammaproteobacteria	o__Xanthomonadales	f__Xanthomonadaceae	g__	s__	
	p__Proteobacteria	c__Betaproteobacteria	o__Burkholderiales	f__Alcaligenaceae	g__	s__	
	p__Proteobacteria	c__Betaproteobacteria	o__Burkholderiales	f__Alcaligenaceae	g__Achromobacter	s__	
**Kekua bao (n=8)**	p__Proteobacteria	c__Alphaproteobacteria	o__Rhizobiales	f__Rhizobiaceae	g__Agrobacterium	s__	32
	p__Proteobacteria	c__Gammaproteobacteria	o__Pseudomonadales	f__Moraxellaceae	g__Enhydrobacter	s__	
	p__Proteobacteria	c__Gammaproteobacteria	o__Enterobacteriales	f__Enterobacteriaceae	g__Erwinia		
	p__Proteobacteria	c__Alphaproteobacteria	o__Rhizobiales	f__Brucellaceae	g__Ochrobactrum	s__	
	p__Proteobacteria	c__Gammaproteobacteria	o__Pseudomonadales	f__Pseudomonadaceae	g__Pseudomonas	s__	
	p__Proteobacteria	c__Gammaproteobacteria	o__Pseudomonadales	f__Pseudomonadaceae	g__Pseudomonas	s__viridiflava	
	p__Proteobacteria	c__Gammaproteobacteria	o__Xanthomonadales	f__Xanthomonadaceae	g__Stenotrophomonas	s__	
	p__Proteobacteria	c__Alphaproteobacteria	o__Rhizobiales				
	p__Proteobacteria	c__Gammaproteobacteria	o__Enterobacteriales	f__Enterobacteriaceae			
